# Associations between inadequate sleep and obesity in the US adult population: analysis of the national health interview survey (1977–2009)

**DOI:** 10.1186/1471-2458-14-290

**Published:** 2014-03-29

**Authors:** Girardin Jean-Louis, Natasha J Williams, Daniel Sarpong, Abhishek Pandey, Shawn Youngstedt, Ferdinand Zizi, Gbenga Ogedegbe

**Affiliations:** 1 Division of Internal Medicine, Center for Healthful Behavior Change, NYU Medical Center, New York, NY, USA; 2 RTRN-DCC, SHS and RCMI-CEH, Jackson State University, Jackson, MS, USA; 3 Department of Family Medicine, SUNY Downstate Medical Center, Brooklyn, NY, USA; 4 Department of Exercise Science, Arnold School of Public Health, University of South Carolina, Columbia, SC, USA; 5 Dorn VA Medical Center, Columbia, SCUSA

**Keywords:** Overweight, Obesity, Short sleep, Long sleep

## Abstract

**Background:**

Epidemiologic studies show a curvilinear relationship between inadequate sleep (< 7 or > 8 hours) and obesity (Body Mass Index > 30 kg/m2), which have enormous public health impact.

**Methods:**

Using data from the National Health Interview Survey, an ongoing nationally representative cross-sectional study of non-institutionalized US adults (≥18 years) (1977 through 2009), we examined the hypothesis that inadequate sleep is independently related to overweight/obesity, with adjustment for socio-demographic, health risk, and medical factors. Self- reported data on health risks, physician-diagnosed medical conditions, sleep duration, and body weight and height were used.

**Results:**

Prevalence of overweight and obesity increased from 31.2% to 36.9% and 10.2% to 27.7%, respectively. Whereas prevalence of very short sleep (<5 hours) and short sleep (5–6 hours) has increased from 1.7% to 2.4% and from 19.7% to 26.7%, it decreased from 11.6% to 7.8% for long sleep. According to multivariate-adjusted multinomial regression analyses, odds of overweight and obesity associated with very short sleep and short sleep increased significantly from 1977 to 2009. Odds of overweight and obesity conferred by long sleep did not show consistent and significant increases over the years. Analyses based on aggregated data showed very short sleepers had 30% greater odds of being overweight or were twice as likely to be obese, relative to 7–8 hour sleepers. Likewise, short sleepers had 20% greater odds of being overweight or 57% greater odds of being obese. Long sleepers had 20% greater odds of being obese, but no greater odds of being overweight.

**Conclusions:**

Our findings support the hypothesis that prevalence of very short and short sleep has gradually increased over the last 32 years. Inadequate sleep was associated with overweight and obesity for each available year.

## Background

Inadequate sleep and obesity are important public health challenges with enormous personal, social, and health care costs [1-4]. The extant literature presents mixed findings regarding association between average sleep duration and body weight. A likely explanation for failure to demonstrate such associations may be that the average, rather than inadequate sleep, as herein defined [very short sleep (<5 hours), short sleep (5–6 hours) or long sleep (>8 hours)], is considered as the exposure variable in those studies. It may also be that varying factors such as measurement errors in eliciting responses about habitual sleep duration may have hampered the ability to establish direct comparisons across published studies [5].

The preponderance of epidemiologic evidence suggests a curvilinear relationship between individuals’ habitual sleep duration and their weight, such that inadequate sleep (short or long) is associated with increased risk of obesity among adults in the United States [1,6-11]. Results of longitudinal studies also demonstrated that inadequate sleep strongly predicts increased body weight [9,10]. A prospective study assessing risk of developing obesity at 1-year among 35,247 respondents sleeping < 5 hours or 5–6 hours found adjusted ORs of 1.91 (95% CI 1.36, 2.67) and 1.50 (95% CI 1.24, 1.80), respectively [12]. On balance, there are data suggesting that sleep duration may not be predictive of excess weight [5,8,13,14].

Although available epidemiologic data do not permit ascertainment of cause and effect relationships, they represent a systematic approach for exploring the potential link between inadequate sleep and obesity at the population level. Of particular interest is the observation that prevalence of short sleep and obesity have increased in the past 40 years. Data from the Alameda County Health and Ways of Living Study show a 72% increase in the prevalence of short sleep (<7 hours) from 1965 to 1999 [15]. Data from the National Health and Nutrition Examination Surveys show a 52% increase in the prevalence of obesity from 1971 to 1999 [15]. Increased prevalence of inadequate sleep seems to parallel increased prevalence of obesity for the same period.

Previous studies exploring associations between inadequate sleep and obesity are largely restricted to single years of observation, preventing ascertainment of important trends, or constrained by differing sampling methodologies and analytic approaches, precluding direct comparisons with available studies. One important limitation relates to the use of differing questions to elicit habitual sleep duration across available studies that may yield biased answers [5,16,17]. Such limitations hamper definitive conclusions about the relationship between increased prevalence of obesity and inadequate sleep. The National Health Interview Survey, which has monitored the health of the United States population since 1957, provides a unique data set to determine associations between inadequate sleep and obesity over an extensive period of time using robust analytic techniques. The first hypothesis tested in this study is that both inadequate sleep and overweight/obesity have gradually increased across the last 32 years. The second hypothesis is that inadequate sleep (short or long) is independently associated with overweight and obesity after adjustment for socio-demographic, health risk, and comorbidity.

## Methods

The National Health Interview Survey is an ongoing, cross-sectional, in-person household interview survey conducted annually by the Centers for Disease Control’s National Center for Health Statistics. The National Health Interview Survey uses a multistage area probability design, sampling non-institutionalized representatives of the civilian population of the United States. Probability samples of the adult population of all 50 states and District of Columbia were obtained. Details on sample design are provided elsewhere [18].

During face-to-face interviews, respondents provided socio-demographic data (age, sex, race/ethnicity, average family income, and education), health risks (smoking status and alcohol intake), and physician diagnosed chronic conditions or diseases (hypertension, diabetes, coronary heart disease, cancer, kidney disease, stroke, and myocardial infarction). Participants estimated habitual sleep duration (using full-hour increments—5 hours, 6 hours, 7 hours etc.) by responding to the following question: “On average, how many hours of sleep do you get in a 24-hour period?” Sleep duration was coded as either very short sleep (< 5 hours/night), short sleep (5–6 hours/night), or long sleep (>8 hours/night), referenced to 7–8 hours/night as healthy sleep [19]. These cut-off points showed significant associations with health risks [20,21]. Body mass index (BMI), which was obtained by computing the ratio of self-reported weight (in kilogram) and height (in meter squared), was coded as overweight (BMI = 25–29.9 kg/m^2^) and obesity (≥ 30 kg/m^2^), referenced to normal weight (BMI = 18.5 - 24.9 kg/m^2^). Participants rated their mood within the last 30 days based on the K-6 scaling system [22]; a score ≥ 13 indicated emotional distress [23]. If participants reported either moderate or vigorous or both, they were classified as having engaged in physical activity (defined as ≥ 150 minutes/week of moderate physical activity or ≥ 75 minutes/week of vigorous activity). The National Health Interview Survey is subject to the Ethics Review Board (ERB) of the Center for Disease Control and Prevention and the National Center for Health Statistics. The data are publicly available.

### Statistical analysis

Analyses were based on National Health Interview Survey data obtained from 1977 through 2009. Since the NHIS dataset includes data from different samples using a multistage area probability sampling design, all analyses performed in this study were based on weighted statistics using the final weights provided with the NHIS dataset. These weights represent a product of weights for corresponding units computed in each of the sampling stages. Once these data were harmonized and standardized across available years, additional processing was unnecessary. As the Center for Disease Control recommends, the complex survey analysis techniques we used accounted for the weights, strata and clusters in the National Health Interview Survey design.

Descriptive analysis was used to ascertain prevalence of variables of interest: overweight/obesity and inadequate sleep across all available years of observation. In order to test the first hypothesis, we used multivariate logistic regression models for complex survey, yielding age and sex-adjusted prevalence of overweight and obesity as well as inadequate sleep (very short sleep, short sleep, and long sleep). Univariate associations of several socio-demographic (race, education, marital status, poverty), health risk (smoking status, alcohol consumption, physical activity, and emotional distress), and medical conditions (prevalent hypertension, diabetes, stroke, heart attack and heart disease) with these factors were also ascertained. To test the second hypothesis, we used multinomial complex survey logistic regression analysis, assessing the associations of inadequate sleep with overweight/obesity, adjusting for effects of covariates [24,25] for each year with adequate data (1977 – 2009). We also used a hierarchical multivariate-adjusted multinomial logistic regression for complex survey designs to obtain eight separate adjusted models. Since data were not available for each year beginning 1977 and ending 2009, a time-lag factor (three-year interval) was created to adjust for potential time effects on observed associations. Thus, the effect of time was determined using three-year increments. This was achieved by first setting 1977 as the reference year and then dividing the length of time from the reference to the years of data collection by three. Hence, the unit change in time was three years. This approach is similar to a unit of change being one standard deviation on the measurement scale. All analyses were performed using Proc Survey logistic--SAS (SAS Institute, Cary, N.C.) [26,27].

## Results

As shown in Table 1, descriptive analyses indicated a trend in the prevalence of inadequate sleep duration (very short, short or long sleep) and overweight/obesity from 1977 to 2009. Specifically, the number of respondents (crude prevalence estimates) classified as overweight or obese increased from 31.2% to 36.9% and 10.2% to 27.7%, respectively. Whereas prevalence of very short sleep (<5 hours) and short sleep (5–6 hours) has increased from 1.7% to 2.4% and from 19.7% to 26.7%, it decreased from 11.6% to 7.8% for long sleep. It is noteworthy that the prevalence of short sleep may have reached a plateau in 2006; with estimates suggesting 27.1% of the United States population were short sleepers.

**Table 1:** Characteristics of adult respondents in the National Health Interview Survey (1977–2009)

**Characteristics**	**1977**	**1983**	**1985**	**1990**	**2004**	**2005**	**2006**	**2007**	**2008**	**2009**
**N**	**18,386**	**9,931**	**28,999**	**35,466**	**24,092**	**24,290**	**18,804**	**18,067**	**16,926**	**21,788**
**Demographics and health risks**										
**Gender, female (%)**	54.7	56.3	51.8	51.3	50.3	50.2	50.0	49.9	50.2	50.4
**Age, years (mean (SD))**	42(15)	40(15)	40(17)	40(17)	42(17)	42(17)	42(18)	42(18)	42(19)	42(19)
**Race/ethnicity (%)**										
*** Whites***	88.6	85.7	85.7	84.0	83.4	83.0	81.5	81.4	81.2	81.0
*** African Americans/Blacks***	9.9	10.4	10.9	11.4	11.6	11.8	12.4	12.2	12.3	12.6
*** Other***	1.5	3.9	3.4	4.7	4.9	5.2	6.1	6.4	6.4	6.4
**Education, ≥ HS (%)**	71.3	76.7	79.3	81.9	87.8	88.0	87.7	88.3	88.4	88.7
**Marital status (%)**										
*** Married***	72.1	67.2	66.9	66.6	53.4	58.9	58.2	57.2	56.4	55.4
*** Widowed, divorced & separated***	13.9	13.8	12.6	13.5	21.8	15.9	16.0	16.1	16.2	16.6
*** Single***	14.0	19.0	20.5	19.9	24.8	25.2	25.8	26.7	27.4	28.0
**Total family income, ≥$ 35 K (%)**	--	22.2	29.8	45.5	64.1	65.4	64.9	68.8	70.4	70.0
**Poverty status, below (%)**	--	8.5	11.2	9.4	10.2	10.4	11.7	11.3	11.6	12.5
**Ever smoked, yes (%)**	58.4	55.1	55.5	50.8	42.4	42.5	41.8	41.4	42.0	42.4
**Alcohol consumption (%)**										
*** Never drinkers***	--	--	--	15.3	22.4	22.1	22.6	21.3	19.0	18.2
*** Former drinkers***	--	--	--	8.9	13.0	12.7	12.9	13.1	12.5	13.3
*** Current drinkers***	--	--	--	75.9	64.6	65.2	64.5	65.6	68.5	68.5
**Normal (%)**	58.6	59.4	56.6	52.6	39.3	38.4	37.7	36.9	36.5	35.4
**Overweight (%)**	31.2	29.1	31.6	33.4	36.9	37.1	36.2	36.8	36.0	36.9
**Obesity (%)**	10.2	11.4	11.8	14.0	23.8	24.6	26.1	26.2	27.5	27.7
**Physical activity (%)**	--	--	--	--	10.9	11.5	11.2	11.5	11.9	12.7
**Emotional distress (%)**	--	--	--	--	3.3	3.0	3.2	2.8	3.4	3.5
***Medical conditions***										
**Diabetes (%)**	--	3.6	--	--	6.3	6.7	7.0	6.8	7.5	8.2
**Hypertension (%)**	--	21.6	21.9	20.7	21.0	21.1	22.8	22.7	24.6	24.1
**Cancer (%)**	--	2.9	--	--	5.0	5.5	5.2	5.5	5.9	6.1
**Coronary heart disease (%)**	--	1.7	--	--	2.7	2.9	2.8	2.9	2.5	3.0
**Myocardial infarction (%)**	--	--	--	--	2.1	2.2	2.2	2.1	2.4	2.2
**Stroke (%)**	--	0.9	1.2	1.3	1.5	1.3	1.5	1.6	1.8	1.6
**Kidney disease (%)**	--	--	--	--	1.3	1.3	1.2	1.1	1.2	1.4
***Sleep measures***										
**Hours of sleep (mean (SD))**	7.4(1.3)	7.3(1.3)	7.4(1.5)	7.3(1.5)	7.1(1.5)	7.1(1.5)	7.1(1.6)	7.1(1.5)	7.1(1.6)	7.1(1.7)
**Sleep duration categories (%)**										
*** Very short sleep***	1.7	2.0	1.3	1.5	2.2	2.1	2.4	2.0	2.4	2.4
*** Short sleep***	19.7	21.0	20.8	23.0	26.8	26.9	27.1	25.4	26.6	26.7
*** Normal sleep***	66.8	67.5	67.1	66.6	63.7	63.6	63.5	65.6	63.5	63.0
*** Long sleep***	11.6	9.5	10.8	8.9	7.2	7.3	7.0	6.9	7.4	7.8

*Abbreviation*: *SD* standard deviation.

As shown in Table 2, analyses based on aggregated data (1977–2009) showed very short sleepers had 30% greater odds of being overweight or were twice as likely to be obese, relative to 7–8 hour sleepers. Likewise, short sleepers had 20% greater odds of being overweight or 57% greater odds of being obese. Long sleepers had 20% greater odds of being obese, but no greater odds of being overweight. Results also suggested that age and sex-adjusted prevalence estimates of overweight/obesity and inadequate sleep for the entire study period were significantly affected by several factors. For overweight and obesity, these included self-reported race/ethnicity as black, having less than high school education, being separated, being below the poverty level, being a former or current smoker, former or current drinker, having a diagnosis of diabetes, hypertension, kidney disease, or coronary heart disease, and having experienced a myocardial infarction or a stroke. For very short, short, and long sleep, these factors included black race/ethnicity, being separated, divorced, or widowed, income below the poverty level, being a current or former smoker or drinker, having a diagnosis of kidney disease, diabetes, hypertension, coronary heart disease, or cancer, and having experienced a myocardial infarction or a stroke. Of note, emotional distress was associated with obesity as well as very short, short, and long sleep, while physical activity was associated with obesity and only long sleep.

**Table 2:** Age-sex-adjusted associations of demographics, health risks, and medical conditions with overweight/obesity and inadequate sleep (1977 – 2009)

**Characteristics**	**Overweight**	**Obesity**	**Very short sleep**	**Short sleep**	**Long sleep**
	**OR (95% CI)**	**OR (95% CI)**	**OR (95% CI)**	**OR (95% CI)**	**OR (95% CI)**
**Demographics**					
**Race/ethnicity (*reference: Whites*)**					
*** Blacks***	1.42 (1.36, 1.47)*	1.98 (1.90, 2.06)*	2.24 (2.04, 2.45)*	1.54 (1.49, 1.60)*	1.62 (1.54, 1.71)*
*** Others***	0.73 (0.69, 0.77)*	0.55 (0.51, 0.60)*	1.05 (0.88, 1.25)*	1.15 (1.09, 1.22)*	0.95 (0.87, 1.05)
**Education (*reference: High School or higher*)**^**‡**^	1.10 (1.06, 1.14)*	1.24 (1.19, 1.29)*	1.93 (1.77, 2.11)*	1.04 (1.00, 1.08)*	2.01 (1.92, 2.10)*
**Marital status (*reference: married*)**					
*** Widowed***	0.92 (0.87, 0.98)*	0.98 (0.91, 1.05)	2.17 (1.87, 2.52)*	1.38 (1.30, 1.47)*	1.58 (1.45, 1.73)*
*** Divorced***	0.96 (0.92, 1.00)	1.12 (1.07, 1.16)*	2.67 (2.41, 2.95)*	1.55 (1.50, 1.61)*	1.16 (1.09, 1.23)
*** Separated***	1.10 (1.02, 1.18)*	1.39 (1.28, 1.50)*	3.53 (3.03, 4.11)*	1.59 (1.48, 1.70)*	1.50 (1.35, 1.66)*
*** Single***	0.81 (0.78, 0.84)*	0.99 (0.95, 1.03)	1.47 (1.32, 1.64)*	1.03 (0.99, 1.06)*	1.59 (1.51, 1.67)*
**Total family income (reference: ≥ $35,000)**	0.89 (0.87, 0.92)*	0.95 (0.92, 0.98)*	2.09 (1.92, 2.27)*	1.04 (1.02, 1.07)*	1.84 (1.75, 1.92)*
**Poverty status (*reference: above poverty*)**	1.05 (1.00, 1.10)*	1.39 (1.32, 1.45)*	2.86 (2.59, 3.17)*	1.19 (1.14, 1.24)*	2.04 (1.93, 2.16)*
**Smoking status (*reference: never smokers*)**	0.99 (0.99, 0.99)*	0.99 (0.99, 0.99)*	1.06 (1.05, 1.06)*	1.02 (1.02, 1.03)*	1.04 (1.03 1.04)*
**Alcohol consumption (*reference: never*)**					
*** Former***	1.09 (1.03, 1.15)*	1.35 (1.27, 1.43)*	1.92 (1.67, 2.21)*	1.53 (1.45, 1.61)*	1.35 (1.25, 1.47)*
*** Current***	0.95 (0.92, 0.99)*	0.86 (0.82, 0.90)*	0.98 (0.88, 1.10)*	1.28 (1.23, 1.33)*	0.82 (0.77, 0.87)*
**Physical activity (reference: yes)**^**a**^	1.05 (0.99, 1.10)	1.32 (1.24, 1.40)*	1.08 (0.99, 1.17)	1.08 (1.03, 1.18)	1.47 (1.32, 1.63)*
**Emotional distress (reference: yes)**^**a**^	1.07 (0.96, 1.18)	1.88 (1.71, 1.35)*	5.51 (5.02, 6.05)*	5.51 (5.02, 6.05)*	3.07 (2.72, 3.46)*
***Self-reported comorbid conditions***					
** Cancer**	1.05 (0.99, 1.10)	1.32 (1.24, 1.40)*	1.50 (1.28, 1.77)*	1.10 (0.99, 1.17)	1.47 (1.32, 1.63)*
** Diabetes**	1.84 (1.69, 1.99)*	4.69 (4.43, 5.05)*	2.15 (1.85, 2.50)*	1.24 (1.16, 1.31)*	1.93 (1.77, 2.11)*
** Hypertension**	1.70 (1.65, 1.76)*	3.66 (3.52, 3.79)*	2.14 (1.95, 2.35)*	1.28 (1.24, 1.32)*	1.52 (1.45, 1.60)*
** Kidney disease**	1.02 (0.87, 1.18)*	1.52 (1.31, 1.77)*	3.10 (2.38, 4.01)*	0.60 (0.52, 0.68)*	1.77 (1.47, 2.13)*
** Myocardial infarction**	1.19 (1.04, 1.35)*	1.96 (1.73, 2.23)*	2.89 (2.33, 2.59)*	1.24 (1.11, 1.38)*	2.54 (2.20, 2.94)*
** Coronary heart disease**	1.27 (1.13, 1.42)*	1.92 (1.72, 2.14)*	2.30 (1.89 2.80)*	1.15 (1.04 1.25)*	2.15 (1.87, 2.44)*
** Stroke**	1.07 (0.96, 1.20)	1.55 (1.38, 1.74)*	4.19 (3.45, 5.09)*	1.39 (1.25, 1.55)*	3.04 (2.67, 3.45)*
***Sleep measure*** **(*reference: healthy sleep*)**					
*** Very short sleep***	1.30 (1.19, 1.43)*	2.17 (1.97, 2.40)*	--	--	--
*** Short sleep***	1.20 (1.16, 1.23)*	1.57 (1.52, 1.63)*	--	--	--
*** Long sleep***	0.99 (0.94, 1.04)	1.20 (1.14, 1.26)*	--	--	--

*Abbreviations*: *CI* confidence interval, *OR* odds ratio.

*P < 0.05.

^a^These measures were available from 2004 to 2009.

^‡^Compares those with less high school education to person with a high school diploma or higher.

As shown in Table 3, increased odds of overweight were associated with very short sleep, with odds ranging from 26% to 47%, but results were significant only for years 2004 and 2007; odds of overweight associated with short sleep ranged from 5% to 36%, with significant findings for all years except for 2005 and 2006. Odds of overweight conferred by long sleep ranged from 1% to 11%, but were not significant for any of the available years. Likewise, increased odds of obesity were associated with very short sleep, with odds ranging from 6% to twice as likely, and results were significant for all years except for 1983 and 2006; odds associated with short sleep ranged from 38% to 88%, with significant findings for all years. Odds of obesity conferred by long sleep ranged from 1% to 26%, but were not significant for any of the available years.

**Table 3:** Multivariate-adjusted associations of very short, short, and long sleep with overweight/obesity

	**Overweight**			**Obesity**		
	**Very short sleep**	**Short sleep**	**Long sleep**	**Very short sleep**	**Short sleep**	**Long sleep**
**Year**	**OR (95% CI)**	**OR (95% CI)**	**OR (95% CI)**	**OR (95% CI)**	**OR (95% CI)**	**OR (95% CI)**
**1983**	1.37 (0.93, 2.02)	1.36 (1.19, 1.54)*	1.02 (0.85, 1.23)	1.34 (0.82, 2.20)	1.38 (1.15, 1.65)*	1.01 (0.78, 1.31)
**1985**	1.29 (0.96, 1.75)	1.18 (1.09, 1.28)*	0.98 (0.86, 1.10)	1.81 (1.29, 2.54)*	1.54 (1.38, 1.73)*	1.08 (0.92, 1.27)
**1990**	0.89 (0.69, 1.16)	1.31 (1.22, 1.41)*	1.09 (0.97, 1.22)	1.43 (1.07, 1.91)*	1.50 (1.37, 1.65)*	1.04 (0.90, 1.21)
**2004**	1.38 (1.02, 1.85)*	1.35 (1.15, 1.58)*	1.11 (0.94, 1.32)	1.92 (1.43, 2.57)*	1.88 (1.59, 2.21)*	1.26 (1.05, 1.51)
**2005**	1.26 (0.92, 1.73)	1.05 (0.93, 1.17)	1.06 (0.87, 1.30)	2.10 (1.54, 2.86)*	1.48 (1.31, 1.68)*	1.19 (0.96, 1.47)
**2006**	1.26 (0.92, 1.73)	1.05 (0.93, 1.17)	1.05 (0.86, 1.29)	1.06 (0.87, 1.30)	1.48 (1.31, 1.68)*	1.19 (0.96, 1.47)
**2007**	1.47 (1.05, 2.05)*	1.18 (1.06, 1.32)*	1.06 (0.87, 1.30)	1.71 (1.21, 2.42)*	1.40 (1.24, 1.58)*	1.21 (0.98, 1.49)
**2008**	1.27 (0.90, 1.80)	1.24 (1.11, 1.39)*	0.87 (0.72, 1.06)	1.53 (1.08, 2.18)*	1.38 (1.23, 1.55)*	0.99 (0.81, 1.21)
**2009**	1.33 (0.97, 1.82)	1.20 (1.09, 1.34)*	1.01 (0.84, 1.22)	1.64 (1.18, 2.30)*	1.55 (1.39, 1.73)*	1.18 (0.97, 1.42)

*Abbreviations*: *CI* confidence interval, *OR* odds ratio.

*P < 0.05.

Covariate adjusted in the model included: age, sex, race/ethnicity, education, marital status, family income, poverty status, history of smoking, alcohol consumption, physical activity, emotional stress, history of diabetes, history of hypertension, history of stroke, and coronary heart disease/myocardial infarction.

Associations between inadequate sleep and overweight/obesity were further ascertained using a hierarchical multivariate-adjusted multinomial regression analysis, yielding 8 distinct models. As depicted in Table 4, the final model showed that very short sleepers had 23% or 59% greater odds of being overweight or obese, and short sleepers had 14% or 42% greater odds of being overweight or obese. Long sleepers had 12% greater odds of being obese, but no greater odds of being overweight; associations for long sleep were not significant in the final model.

**Table 4:** Hierarchical multivariate-adjusted associations of very short, short, and long sleep with overweight/obesity

**Characteristics**	**Model 1**^**a**^		**Model 2**^**b**^		**Model 3**^**c**^	
	**Overweight**	**Obesity**	**Overweight**	**Obesity**	**Overweight**	**Obesity**
***Sleep measures***	**OR (95% CI)**	**OR (95% CI)**	**OR (95% CI)**	**OR (95% CI)**	**OR (95% CI)**	**OR (95% CI)**
Reference (7–8 hours)	1.00	1.00	1.00	1.00	1.00	1.00
Very short sleep	1.26 (1.14, 1.38)*	2.05 (1.85, 2.26)*	1.29 (1.16, 1.43)*	1.93 (1.73, 2.16)*	1.30 (1.16, 1.47)*	1.95 (1.73, 2.20)*
Short sleep	1.17 (1.14, 1.20)*	1.51 (1.46, 1.56)*	1.19 (1.15, 1.23)*	1.52 (1.46, 1.58)*	1.20 (1.15, 1.24)*	1.53 (1.47, 1.60)*
Long sleep	1.03 (0.98, 1.08)	1.30 (1.23, 1.37)*	1.03 (0.97, 1.09)*	1.23 (1.15, 1.31)*	1.04 (0.98, 1.11)	1.24 (1.16, 1.33)*
**Characteristics**	**Model 4**^**d**^		**Model 5**^**e**^		**Model 6**^**f**^	
	**Overweight**	**Obesity**	**Overweight**	**Obesity**	**Overweight**	**Obesity**
***Sleep measures***	**OR (95%CI)**	**OR (95%CI)**	**OR (95%CI)**	**OR (95%CI)**	**OR (95%CI)**	**OR (95%CI)**
Reference (7–8 hours)	1.00	1.00	1.00	1.00	1.00	1.00
Very short sleep	1.25 (1.11, 1.41)*	1.72 (1.53, 1.95)*	1.34 (1.18, 1.53)*	1.91 (1.68, 2.18)*	1.36 (1.19, 1.55)*	1.99 (1.75, 2.27)*
Short sleep	1.18 (1.14, 1.23)*	1.48 (1.42, 1.55)*	1.17 (1.12, 1.22)*	1.51 (1.44, 1.58)*	1.18 (1.13, 1.23)*	1.51 (1.45, 1.58)*
Long sleep	1.02 (0.95, 1.09)	1.16 (1.08, 1.25)	1.02 (0.95, 1.10)	1.20 (1.11, 1.29)*	1.03 (0.95, 1.11)	1.24 (1.15, 1.34)*
**Characteristics**	**Model 7**^**g**^		**Model 8**^**h**^			
	**Overweight**	**Obesity**	**Overweight**	**Obesity**		
***Sleep measures***	**OR (95%CI)**	**OR (95%CI)**	**OR (95%CI)**	**OR (95%CI)**		
Reference (7–8 hours)	1.00	1.00	1.00	1.00		
Very short sleep	1.30 (1.15, 1.46)*	1.94 (1.72, 2.19)*	1.23 (1.08, 1.40)*	1.59 (1.40, 1.81)*		
Short sleep	1.20 (1.15, 1.24)*	1.53 (1.47, 1.60)*	1.14 (1.09, 1.19)*	1.42 (1.36, 1.49)*		
Long sleep	1.04 (0.98, 1.11)	1.24 (1.15 1.33)*	0.99 (0.92, 1.07)	1.12 (1.03, 1.21)		

*Abbreviations*: *CI* confidence interval, *OR* odds ratio; *P < 0.05.

^**a**^*Model 1: age-sex-adjusted independent associations of inadequate sleep (short and long) with obesity.*

^**b**^*Model 2: Model 1 plus demographic factors.*

^**c**^*Model 3: Model 2 plus smoking and alcohol consumption.*

^**d**^*Model 4: Model 3 plus hypertension.*

^**e**^*Model 5: Model 3 plus diabetes.*

^**f**^*Model 6: Model 3 plus and c*oronary heart disease*/myocardial infarction.*

^**g**^*Model 7: Model 3 plus stroke.*

^**h**^*Model 8: Parsimonious Model (covariates adjusted in the model included: age, sex, race/ethnicity, education, total family income, smoking status, history of diabetes, and history of hypertension).*

## Discussion

Emerging consensus suggests that individuals sleeping 7–8 hours may be at reduced risks of medical comorbidity and early mortality, compared with short and long sleepers [1,28-31]. However, it is much less certain whether the prevalence of inadequate sleep (very short, short, and long sleep) among American adults has increased over the years and what the effects of such increases might be. Using data from the National Health Interview Survey, we examined 32-year trend in the prevalence of inadequate sleep duration and whether such trends were associated with observed increases in the prevalence of overweight and obesity in the adult population of the United States. Our main findings support the hypothesis that the prevalence of insufficient sleep (very short or short sleep) has increased over the last 32 years with 1.7 of Americans being very short sleepers in 1977 compared with 2.4% in 2009 and 19.7% of Americans being short sleepers in 1977 compared with 26.7% in 2009.

Of interest was the comparability of our prevalence estimates for short sleepers (< 7 hours) to those derived from the Sleep Heart Health Study, which was 27.5% in 2005 [32]. Prevalence of short sleep in 1977 was lower than that reported in the data from Alameda County Health and Ways of Living Study, which revealed that 15.2% of respondents slept less than 7 hours in 1965 [15]. Thus, our observation suggests nearly a two-fold increase in the prevalence of short sleepers among Americans, which if replicated in other national datasets, would have an important public health impact. Although fewer Americans reported very short sleep (< 5 hours), it is noteworthy that the prevalence of very short sleep also increased during the same time span. Thus, studies classifying individuals into these two categories may reveal critical health-related outcomes that would be important targets for interventions.

Our findings are consistent with a recent review of trends in sleep duration observed from epidemiologic sleep data in the United States [33]. Accordingly, the preponderance of evidence suggests that United States adults have been sleeping less and less, with the modal sleep duration decreasing from 1979 to 1998 by over one hour [30,33]. Using a different criterion for defining short sleep (<6 hours), investigators noted an increase in short sleep from 7.6% in 1975 to 9.3% in 2006 [34]. Evidently, discrepancy in these two analyses relates to differing definitions of short sleep, which should be considered when comparing prevalence estimates of short sleep across epidemiologic studies [29]. Notwithstanding this discrepant finding, trends observed in both studies are similar, thus supporting the notion that the number of American short sleepers has increased in the last 40 years.

It is noteworthy that the prevalence of short sleep may have reached a plateau in 2006 when 27.1% of United States adults were short sleepers compared with 26.7% in 2009. This observation could be important, but has not been fully elucidated. A similar observation was noted in two previous analyses based on other United States samples. While the first one focusing on data obtained between 1985 and 2006 revealed an increase in the prevalence of short sleep [35], the second one, gathered between 2004 and 2007, showed a decrease in prevalence estimates [36]. It is of interest to examine whether trends observed in our analyses of the National Health Interview Survey data from 1977 to 2009 could be replicated in other epidemiologic sleep data. Future epidemiologic sleep studies are necessary to document any sustained reversal in these observed trends.

If indeed the prevalence of short sleep has ceased to increase, factors underlying this effect should be emphasized in public health campaigns promoting healthy sleep. Plausibly, such factors may provide important clues to help clarify mixed findings in the literature regarding increases in the prevalence of short sleepers over time. Review of the literature suggests that studies that have failed to demonstrate a consistent increase in the number of short sleepers in the United States population have utilized data spanning only the last decade, which is precisely the interval within which the dip seems to have occurred [33]. Those relying on a much longer period of observations have tended to show consistent and gradual increases in the prevalence of short sleep [29,33], as we demonstrated in the present analyses.

Contrary to previous studies that focused only on trends in short sleep, we also ascertained trends in long sleep. As expected, the unadjusted prevalence of long sleep appears to have decreased from 11.6% to 7.8%, which is commensurate with relative increases in the prevalence of very short and short sleep. This could simply be a reflection of gradual decline in sleep duration, affecting all strata of the United States population, as has been demonstrated in previous studies. In effect, examination of data obtained from 1959 to 2005 reveals a decrease in modal sleep duration of approximately 1.5 hours [15,29,30,37]. Epidemiologic data, which have consistently shown a U-shaped association of sleep duration with mortality/morbidity, suggest that whereas increased prevalence of short sleep might have negative effects, reductions in long sleep might have many positive effects [38].

The finding of an increased prevalence of obesity in the US population is consistent with several published papers using other well-characterized epidemiologic studies [3,39]. Specifically, crude prevalence estimates of overweight and obesity increased from 31.2% and 10.2% in 1977 to 36.9% and 27.7% in 2009. Of note, whereas the prevalence of very short and short sleep might have slightly declined after 2007 in the present data, the prevalence of overweight and obesity has not decreased at all. That the continued rise in overweight and obesity from 2004–2009 occurred in the absence of an increase in short sleep duration during the same time period reinforces that the obesity epidemic is probably attributable to multiple other factors, as well as perhaps changes in sleep duration. Of note, a more recent analysis of National Health and Nutrition Examination Survey 2005–2010 data obtained from 13,742 participants (ages ≥ 20 years) found that short sleepers were more likely to be obese and have abdominal obesity [40].

Although cross-sectional epidemiologic data cannot address causality, the purpose of our analyses was to determine whether inadequate sleep and overweight/obesity were independently associated. The first analytic model ascertained age and sex-adjusted prevalence estimates of inadequate sleep and overweight/obesity for the entire study period (1977–2009), while adjusting potential effects of three clusters of confounders (socio- demographics, health risks, and medical diseases). Such adjustments were necessary because preliminary analyses demonstrated that many of these factors have changed over time (Table 1), with potential adverse effects on both inadequate sleep and overweight/obesity [13,15,29]. Initial selection of specific confounders was guided by results of the Alameda County study showing strong associations with inadequate sleep [15]. A recent review paper provides a comprehensive list of the factors potentially affecting associations between inadequate sleep and obesity [29].

Most of the factors that have contributed to increased odds of overweight/obesity also exhibited positive associations with inadequate sleep (very short, short, or long sleep). These results underlie the inherent complexity in delineating potential effects of these factors on associations between inadequate sleep and overweight/obesity, which have also changed over time. Intervention aimed at manipulating sleep to modify risk of becoming overweight or obese may require a careful consideration of all of these factors [13,29,41].

Our multivariate-adjusted regression analyses showed that inadequate sleep was associated with overweight and obesity for each year providing adequate data. Results are consistent with multiple well-characterized, cross-sectional studies evidencing the short sleep-obesity link in the adult population [7,9-11,28,42-45]. Indeed, compared with healthy adult sleepers (7–8 hours) a recent meta-analysis showed that the pooled odds ratio for obesity conferred by short sleep was 55% [28]. That we observed smaller odds for obesity may be a function of more adequate adjustment for confounders in our analyses. In all, these epidemiologic findings are in accordance with several studies showing adverse effects of short sleep on body weight [46-49].

Consistent with emerging interest in studying negative effects of long sleep [50], our analyses revealed increased odds of obesity conferred by long sleep; of interest, odds were not greater for overweight. The fact that odds ratios observed for each year were not significant may be explained by reduced cell sizes due to a relatively smaller number of long sleepers and adjustment for multiple factors. By contrast with other studies, 38% of long sleepers were likely to be overweight or obese in the Quebec Family study [51]. Evidently, that study captured a greater proportion of individuals as their outcome variable aggregated both overweight and obesity. Notwithstanding such differences, these findings demonstrate that long sleep may also be a modifiable risk factor, but the evidence linking long sleep with obesity has generally been less robust and consistent than the evidence linking short sleep with obesity. We should note that a recent study analyzing 38 nationally representative time-use surveys conducted across 10 industrialized countries suggests that long sleep might be more widespread than short sleep duration [14], supporting the notion that long sleep may be equally as important as short sleep in devising public health policies.

The major strengths of our study include the use of epidemiologic data from a well-characterized and nationally representative survey of US adults providing adequate data from 1977 to 2009. This has allowed the determination of secular trends of inadequate sleep as well as overweight and obesity and assessment of independent associations between these health indices. Notably, the study has some limitations. First, although our multivariate models adjusted effects of important confounders, causality could not be established as the exposure (inadequate sleep) and outcome (overweight/obesity) measures were collected concurrently. We acknowledge the possibility of bi-directionality in our epidemiologic approach [52], but available experimental evidence supports the hypothesis that inadequate sleep is likely a ‘sufficient cause’ of obesity [53]. Obesity in and of itself does not seem to make substantial contribution to inadequate sleep, as evidence suggests no difference in sleep parameters between obese and non-obese individuals without comorbidity [54]. Second, derivation of inadequate sleep and overweight/obesity was performed on the basis of self-reported data, which may on occasion be subject to varying biases (e.g., poor recall, social desirability, and forward telescoping). We are aware that subjective reports of sleep duration are often overestimated, relative to objectively determined sleep durations [55]. However, we are not aware of any representative studies that have utilized objective sleep recording devices (e.g., polysomnography or actigraphy) permitting analyses of sleep trends in the United States population over a 32-year period [29,41]. Likewise, use of advanced techniques (e.g., bioelectrical impedance, skin-fold thickness, densitometry, computerized tomography, or magnetic resonance imaging) to assess obesity is not feasible in the field [29,41]. We note that self-reported sleep and weight measures correspond well with objective data. Correlation coefficients between subjective and actigraphic sleep durations ranged from 0.57 to 0.78 [56-58]; correlation coefficient between subjective and measured weight was 0.96 [59]. Third, important factors such as sleep apnea, insomnia, and excessive daytime sleepiness or fatigue, which have adverse effects on sleep duration, were not adjusted in our models; such data were not available in the National Health Interview Survey data. However, it bears noting that when previous analyses adjusted effects of such factors, associations between inadequate sleep and obesity remained virtually unaffected [28,29,41] Future studies should explore the potential influence of various technological advances (e.g., 24-hour cable television programming, internet use, and smart phones) that have occurred over the last two decades on sleep curtailment and their direct or indirect effect on obesity.

## Conclusions

While our analyses were performed on a large dataset with adequate statistical control, it is premature to offer a definitive conclusion regarding causal associations between sleep duration and overweight/obesity. Our study supports the growing evidence that inadequate sleep duration may be an independent risk factor of overweight/obesity. It also appears that the prevalence of overweight/obesity has occurred in tandem with changes in inadequate sleep, which has increased over the last 32 years, although a plateau may have been reached. The findings demonstrate that sleep duration is one domain that should be considered in public health campaigns geared towards decreasing cardio-metabolic risk. Although the obesity-sleep relationship is not entirely understood, considerations should be given to the underlying mechanisms including biological and physiological processes. Physiologic evidence suggests short sleep may influence weight gain through effects on appetite, physical activity, and/or thermoregulation [45]. Care must be exercised when interpreting epidemiologic self-reported sleep data, as varying factors such as measurement errors, response bias, and reverse causation may confound generalizability of the findings. Since we used data from one single source, it is likely that our analyses of subjective data, as captured in the NHIS, would be less biased than analyses comparing results from studies with differing designs and sampling strategies [5,16,17].

## Competing interests

The authors declare that they have no competing interests.

## Authors’ contributions

JLG, WNJ, SD, YS, ZF, PA, and OG developed the conceptual model. JLG, WNJ, SD, YS, OG contributed to the results and the discussion section. JLG, YS, OG drafted the manuscript. All authors read and approved the final manuscript.

## Pre-publication history

The pre-publication history for this paper can be accessed here:

http://www.biomedcentral.com/1471-2458/14/290/prepub

## References

[B1] Youngstedt SD Jean-Louis G Long sleep a greater mortality risk than short sleep in older adults J Am Geriatr Soc 2011 59 957 958 10.1111/j.1532-5415.2011.03396.x 21568977 PMC4792261

[B2] Ogden CL Lamb MM Carroll MD Flegal KM Obesity and socioeconomic status in adults: United States 2005–2008 NCHS Data Brief 2010 50 1 8 21211165

[B3] Flegal KM Carroll MD Kuczmarski RJ Johnson CL Overweight and obesity in the United States: prevalence and trends, 1960–1994 Int J Obes Relat Metab Disord 1998 22 39 47 10.1038/sj.ijo.0800541 9481598

[B4] Rosekind MR Gregory KB Mallis MM Brandt SL Seal B Lerner D The cost of poor sleep: workplace productivity loss and associated costs J Occup Environ Med 2010 52 91 98 10.1097/JOM.0b013e3181c78c30 20042880

[B5] Kurina LM McClintock MK Chen JH Waite LJ Thisted RA Lauderdale DS Sleep duration and all-cause mortality: a critical review of measurement and associations Ann Epidemiol 2013 23 361 370 10.1016/j.annepidem.2013.03.015 23622956 PMC3660511

[B6] Patel SR Zhu X Storfer-Isser A Mehra R Jenny NS Tracy R Redline S Sleep duration and biomarkers of inflammation Sleep 2009 32 200 204 19238807 10.1093/sleep/32.2.200 PMC2635584

[B7] Patel SR Hu FB Short sleep duration and weight gain: a systematic review Obesity (Silver Spring) 2008 16 643 653 10.1038/oby.2007.118 18239586 PMC2723045

[B8] Marshall NS Glozier N Grunstein RR Is sleep duration related to obesity? A critical review of the epidemiological evidence Sleep Med Rev 2008 12 289 298 10.1016/j.smrv.2008.03.001 18485764

[B9] Hasler G Buysse DJ Klaghofer R Gamma A Ajdacic V Eich D Rössler W Angst J The association between short sleep duration and obesity in young adults: a 13-year prospective study Sleep 2004 27 661 666 15283000 10.1093/sleep/27.4.661

[B10] Gangwisch JE Malaspina D Boden-Albala B Heymsfield SB Inadequate sleep as a risk factor for obesity: analyses of the NHANES I Sleep 2005 28 1289 1296 16295214 10.1093/sleep/28.10.1289

[B11] Buxton OM Marcelli E Short and long sleep are positively associated with obesity, diabetes, hypertension, and cardiovascular disease among adults in the United States Soc Sci Med 2010 71 1027 1036 10.1016/j.socscimed.2010.05.041 20621406

[B12] Watanabe M Kikuchi H Tanaka K Takahashi M Association of short sleep duration with weight gain and obesity at 1-year follow-up: a large-scale prospective study Sleep 2010 33 161 167 20175399 10.1093/sleep/33.2.161 PMC2817903

[B13] Stranges S Dorn JM Shipley MJ Kandala NB Trevisan M Miller MA Donahue RP Hovey KM Ferrie JE Marmot MG Cappuccio FP Correlates of short and long sleep duration: a cross-cultural comparison between the United Kingdom and the United States: the Whitehall II Study and the Western New York Health Study Am J Epidemiol 2008 168 1353 1364 10.1093/aje/kwn337 18945686 PMC2727192

[B14] Bin YS Marshall NS Glozier N Sleeping at the limits: the changing prevalence of short and long sleep durations in 10 countries Am J Epidemiol 2013 177 826 833 10.1093/aje/kws308 23524039

[B15] Stamatakis KA Kaplan GA Roberts RE Short sleep duration across income, education, and race/ethnic groups: population prevalence and growing disparities during 34 years of follow-up Ann Epidemiol 2007 17 948 955 10.1016/j.annepidem.2007.07.096 17855122 PMC2140008

[B16] Dowd JB Todd M Does self-reported health bias the measurement of health inequalities in U.S. adults? Evidence using anchoring vignettes from the Health and Retirement Study J Gerontol B Psychol Sci Soc Sci 2011 66 478 489 21666144 10.1093/geronb/gbr050

[B17] Ulander M Arestedt K Svanborg E Johansson P Brostrom A The fairness of the Epworth sleepiness scale: two approaches to differential item functioning Sleep Breath 2013 17 157 165 10.1007/s11325-012-0664-8 22367404

[B18] Botman SL Moore TF Moriarity CL Parsons VL Design and estimation for the National Health Interview Survey, 1995–2004. National Center for Health Statistics Vital Health Stat 2000 130

[B19] National Institutes of Health Your Guide to Healthy Sleep[http://www.nhlbi.nih.gov/health/public/sleep/yg_slp.htm]

[B20] Kripke DF Garfinkel L Wingard DL Klauber MR Marler MR Mortality associated with sleep duration and insomnia Arch Gen Psychiatry 2002 59 131 136 10.1001/archpsyc.59.2.131 11825133

[B21] Tamakoshi A Ohno Y Self-reported sleep duration as a predictor of all- cause mortality: results from the JACC study, Japan Sleep 2004 27 51 54 14998237

[B22] Kessler RC Barker PR Colpe LJ Epstein JF Gfroerer JC Hiripi E Howes MJ Normand SL Manderscheid RW Walters EE Zaslavsky AM Screening for serious mental illness in the general population Arch Gen Psychiatry 2003 60 184 189 10.1001/archpsyc.60.2.184 12578436

[B23] Kessler RC Green JG Gruber MJ Sampson NA Bromet E Cuitan M Furukawa TA Gureje O Hinkov H Hu CY Lara C Lee S Mneimneh Z Myer L Oakley-Browne M Posada-Villa J Sagar R Viana MC Zaslavsky AM Screening for serious mental illness in the general population with the K6 screening scale: results from the WHO World Mental Health (WMH) survey initiative Int J Methods Psychiatr Res 2010 19 Suppl 1 4 22 20527002 10.1002/mpr.310 PMC3659799

[B24] Reyner LA Horne JA Reyner A Gender- and age-related differences in sleep determined by home- recorded sleep logs and actimetry from 400 adults Sleep 1995 18 127 134 7792492

[B25] Unruh ML Redline S An MW Buysse DJ Nieto FJ Yeh JL Newman AB Subjective and objective sleep quality and aging in the sleep heart health study J Am Geriatr Soc 2008 56 1218 1227 10.1111/j.1532-5415.2008.01755.x 18482295

[B26] Research Triangle Institute SUDAAN user’s Manual, Release 9.0. Research Triangle Park 2004 NC: Research Triangle Institute

[B27] SAS Institute Inc SAS/STAT® 9.2. User’s Guide 2008 Cary, NC: SAS Institute Inc. 2008 Ref Type: Internet Communication

[B28] Cappuccio FP Taggart FM Kandala NB Currie A Peile E Stranges S Meta-analysis of short sleep duration and obesity in children and adults Sleep 2008 31 619 626 18517032 10.1093/sleep/31.5.619 PMC2398753

[B29] Nielsen LS Danielsen KV Sorensen TI Short sleep duration as a possible cause of obesity: critical analysis of the epidemiological evidence Obes Rev 2011 12 78 92 10.1111/j.1467-789X.2010.00724.x 20345429

[B30] Jean-Louis G Kripke DF Ancoli-Israel S Klauber M Sepulveda RS Sleep duration, illumination, and activity patterns in a population sample: Effects of gender and ethnicity Biol Psychiatry 2000 47 921 927 10.1016/S0006-3223(99)00169-9 10807965

[B31] Hublin C Partinen M Koskenvuo M Kaprio J Sleep and mortality: a population-based 22-year follow-up study Sleep 2007 30 1245 1253 17969458 10.1093/sleep/30.10.1245 PMC2266277

[B32] Gottlieb DJ Redline S Nieto FJ Baldwin CM Newman AB Resnick HE Punjabi NM Association of usual sleep duration with hypertension: the Sleep Heart Health Study Sleep 2006 29 1009 1014 16944668 10.1093/sleep/29.8.1009

[B33] Bin YS Marshall NS Glozier N Secular trends in adult sleep duration: a systematic review Sleep Med Rev 2012 16 223 230 10.1016/j.smrv.2011.07.003 22075214

[B34] Knutson KL Van CE Rathouz PJ DeLeire T Lauderdale DS Trends in the prevalence of short sleepers in the USA: 1975–2006 Sleep 2010 33 37 45 20120619 10.1093/sleep/33.1.37 PMC2802246

[B35] QuickStats Percentage of Adults Aged >18 Years Who Reported an Average of <6 Hours of Sleep† per 24-Hour Period, by Sex and Age Group 2012 United States: United States, 1985 and 2006 Ref Type: Internet Communication

[B36] Luckhaupt SE Tak S Calvert GM The prevalence of short sleep duration by industry and occupation in the National Health Interview Survey Sleep 2010 33 149 159 20175398 10.1093/sleep/33.2.149 PMC2817902

[B37] Schoenborn CA Health habits of U.S. adults, 1985: the “Alameda 7” revisited Public Health Rep 1986 101 571 580 3097736 PMC1477675

[B38] Youngstedt SD Kripke DF Long sleep and mortality: rationale for sleep restriction Sleep Med Rev 2004 8 159 174 10.1016/j.smrv.2003.10.002 15144959

[B39] Ogden CL Carroll MD Curtin LR McDowell MA Tabak CJ Flegal KM Prevalence of overweight and obesity in the United States, 1999–2004 JAMA 2006 295 1549 1555 10.1001/jama.295.13.1549 16595758

[B40] Ford ES Li C Wheaton AG Chapman DP Perry GS Croft JB Sleep duration and body mass index and waist circumference among US adults Obesity (Silver Spring) 2013 22 598 607 23836704 10.1002/oby.20558 PMC4580243

[B41] Magee CA Iverson DC Huang XF Caputi P A link between chronic sleep restriction and obesity: methodological considerations Public Health 2008 122 1373 1381 10.1016/j.puhe.2008.05.010 18722633

[B42] Kohatsu ND Tsai R Young T Vangilder R Burmeister LF Stromquist AM Merchant JA Sleep duration and body mass index in a rural population Arch Intern Med 2006 166 1701 1705 10.1001/archinte.166.16.1701 16983047

[B43] Taheri S Lin L Austin D Young T Mignot E Short sleep duration is associated with reduced leptin, elevated ghrelin, and increased body mass index PLoS Med 2004 1 e62 10.1371/journal.pmed.0010062 15602591 PMC535701

[B44] Singh M Drake C Roehrs T Hudgel DW Roth T The association between obesity and short sleep duration: a population-based study J Clin Sleep Med 2005 1 357 363 17564401 10.5664/jcsm.26361 PMC13109519

[B45] Vorona RD Winn MP Babineau TW Eng BP Feldman HR Ware JC Overweight and obese patients in a primary care population report less sleep than patients with a normal body mass index Arch Intern Med 2005 165 25 30 10.1001/archinte.165.1.25 15642870

[B46] Shlisky JD Hartman TJ Kris-Etherton PM Rogers CJ Sharkey NA Nickols- Richardson SM Partial sleep deprivation and energy balance in adults: an emerging issue for consideration by dietetics practitioners J Acad Nutr Diet 2012 112 1785 1797 10.1016/j.jand.2012.07.032 23102177

[B47] Goel N Rao H Durmer JS Dinges DF Neurocognitive consequences of sleep deprivation Semin Neurol 2009 29 320 339 10.1055/s-0029-1237117 19742409 PMC3564638

[B48] Banks S Dinges DF Behavioral and physiological consequences of sleep restriction J Clin Sleep Med 2007 3 519 528 17803017 PMC1978335

[B49] Van Dongen HP Maislin G Mullington JM Dinges DF The cumulative cost of additional wakefulness: dose–response effects on neurobehavioral functions and sleep physiology from chronic sleep restriction and total sleep deprivation Sleep 2003 26 117 126 12683469 10.1093/sleep/26.2.117

[B50] Bliwise DL Young TB The parable of parabola: what the U-shaped curve can and cannot tell us about sleep Sleep 2007 30 1614 1615 18246971 10.1093/sleep/30.12.1614 PMC2276137

[B51] Chaput JP Despres JP Bouchard C Tremblay A The association between sleep duration and weight gain in adults: a 6-year prospective study from the Quebec Family Study Sleep 2008 31 517 523 18457239 10.1093/sleep/31.4.517 PMC2279744

[B52] Vgontzas AN Bixler EO Basta M Obesity and sleep: a bidirectional association? Sleep 2010 33 573 574 20469796 10.1093/sleep/33.5.573 PMC2864869

[B53] Rothman KJ Causes. 1976 Am J Epidemiol 1995 141 90 95 7817976 10.1093/oxfordjournals.aje.a117417

[B54] Vgontzas AN Lin HM Papaliaga M Calhoun S Vela-Bueno A Chrousos GP Bixler EO Short sleep duration and obesity: the role of emotional stress and sleep disturbances Int J Obes (Lond) 2008 32 801 809 10.1038/ijo.2008.4 18253159

[B55] Bixler E Sleep and society: an epidemiological perspective Sleep Med 2009 10 S3 6 19660985 10.1016/j.sleep.2009.07.005

[B56] Lockley SW Skene DJ Arendt J Comparison between subjective and actigraphic measurement of sleep and sleep rhythms J Sleep Res 1999 8 175 183 10.1046/j.1365-2869.1999.00155.x 10476003

[B57] Lauderdale DS Knutson KL Yan LL Liu K Rathouz PJ Self-reported and measured sleep duration: how similar are they? Epidemiology 2008 19 838 845 10.1097/EDE.0b013e318187a7b0 18854708 PMC2785092

[B58] O’Donoghue GM Fox N Heneghan C Hurley DA Objective and subjective assessment of sleep in chronic low back pain patients compared with healthy age and gender matched controls: a pilot study BMC Musculoskelet Disord 2009 10 122 10.1186/1471-2474-10-122 19799778 PMC2765952

[B59] Willett W Stampfer MJ Bain C Lipnick R Speizer FE Rosner B Cramer D Hennekens CH Cigarette smoking, relative weight, and menopause Am J Epidemiol 1983 117 651 658 6859020 10.1093/oxfordjournals.aje.a113598

